# Enhancing Outpatient Clinic Recruitment: A Quality Improvement Strategy for a Prospective Research Study

**DOI:** 10.1016/j.arrct.2025.100539

**Published:** 2025-10-28

**Authors:** Priya D. Bolikal, Ryan Schuetter, Tess Guzman, Richa Patel, Paul J. Gubanich, Shari L. Wade, Lynn Babcock, Megan Narad, Sarah M. Eickmeyer, Brad G. Kurowski

**Affiliations:** a Division of Pediatric Rehabilitation Medicine, Cincinnati Children’s, Cincinnati, OH; b Department of Pediatrics, University of Cincinnati College of Medicine, Cincinnati, OH; c Department of Neurology & Rehabilitation Medicine, University of Cincinnati College of Medicine, Cincinnati, OH; d Division of Sports Medicine, Cincinnati Children’s, Cincinnati, OH; e Division of Emergency Medicine, Cincinnati Children’s, Cincinnati, OH; f Division of Behavioral Medicine and Clinical Psychology, Cincinnati Children’s Hospital, Cincinnati, OH; g Department of Physical Medicine & Rehabilitation, University of Kansas School of Medicine, Kansas City, KS; h James M. Anderson Center for Health Systems Excellence, Cincinnati Children's, Cincinnati, OH

**Keywords:** Brain injury, Clinical recruitment, Concussion, Rehabilitation

## Abstract

**Objective:**

To improve study recruitment by increasing the number of eligible participants who receive study information during their outpatient clinical care.

**Design:**

Quality improvement study.

**Setting:**

Outpatient clinics from July 2024 to January 2025.

**Participants:**

Eligible patients across 16 outpatient clinics (N=231, mean age=14.1 ± 2.0 y, women=46%, White=74%).

**Interventions:**

Using quality improvement methodology, the team identified barriers and key drivers for recruitment from clinics. The initial interventions included designating and engaging a clinical staff member in each clinic to distribute study information (recruitment champion), notifying them of potentially eligible patients, tracking progress, and incentivizing recruitment with small rewards. Recruitment was tracked and reviewed monthly to assess progress and identify interventions for ongoing iterations.

**Main Outcome Measures:**

Primary: Percentage of eligible patients who received study information during their clinic visit. Secondary: Study enrollment rates.

**Results:**

During this period, 58.9% of eligible patients received study information during their clinic visits (baseline rate from prior similar study=32.6%). Enrollment from clinic contact averaged 59% (baseline rate from prior similar study=21.8%). Chi-square test indicated a significantly higher proportion of patients received information about the study from clinic staff compared with the prior similar study (χ^2^=36.06, *P*<.001), and those receiving information about the study in clinic corresponded with a higher rate of enrollment compared with the group that did not receive information in clinic (χ^2^=21.33, *P*<.001).

**Conclusions:**

This project demonstrates the successful application of quality improvement methodology toward optimizing clinical recruitment. Approaches used in this study can potentially be generalized to other outpatient clinical populations and research studies.

Recruitment and enrollment of participants from clinical arenas into research studies can be universally challenging.^1^^,^^2^ Underenrollment is often the primary reason that clinical research studies are unable to address their proposed aims.^3^^,^^4^ This challenge stems from a variety of factors, including limited access to eligible participants, time constraints for clinical staff, and a lack of effective communication between research teams and health care providers.^5^^,^^6^ Recruitment of participants for prospective studies is critical for the success of the research studies and the generalizability of data. Without adequate participant representation, research studies risk bias, reduced statistical power, and decreased applicability of findings to broader populations.^2^^,^^7^ Previous work highlights the need for engaging and regular communication of the research team, development of partnerships with clinical teams, and adequate resources to perform recruitment activities as key components in successful recruiting.^6^, ^7^, ^8^

Like many other departments, our own division of pediatric rehabilitation medicine has not established a standardized strategy for clinical recruitment. In a recent research study conducted by our team examining school re-entry of patients aged 5-18 years who sustained mild traumatic brain injuries (mTBIs), 32.6% of eligible patients were informed about the study during their outpatient clinic visits, and 21.8% of eligible patients seen in outpatient clinics enrolled in the study. Because of existing clinic responsibilities, variable staffing models, and already complex clinic workflows, research recruitment has proven not to be a simple, seamless addition for clinical staff. In previous studies, research staff have shared information about research studies with clinical providers and asked that they assist with recruitment, but no standardized workflow was implemented to support clinical research recruitment. We proposed that enhancing collaboration between research teams and clinic staff, as well as clinic-friendly recruitment strategies, would increase recruitment success.

Recruitment barriers have been shown to stem from workflow constraints, communication gaps between clinical and research teams, and competing clinical priorities.^9^ We sought to evaluate the presence and magnitude of these barriers within our own clinical environment in relation to an initiated and unpublished clinical research study (Development of a Mental health Outcomes Screening Tool after mild traumatic brain injury in adolescents: MOST-mTBI) that primarily recruited and enrolled participants from outpatient concussion/mTBI clinics. Potentially eligible participants for this study included English or Spanish speakers aged 11-17 years at the time of injury who sustained a mTBI/concussion within the 3 weeks prior. Patients were not eligible for the study if they were outside the age range, were not English or Spanish speakers, had a history of significant preinjury cognitive impairment, or were without evidence of a traumatic injury. We engaged research and clinical teams early in the process to plan interventions that would target potential barriers with the ultimate goal of increasing the distribution of study information to eligible patients during their clinic visits. This quality improvement (QI) study aimed to: (1) provide study information to 90% of eligible patients seen in outpatient clinics and (2) achieve a study enrollment rate of 50% of eligible patients.

## Methods

This QI study was written and conducted using Standards for Quality Improvement Reporting Excellence (SQUIRE) 2.0 Guidelines.^10^^,^^11^

Recruitment occurred at a tertiary care pediatric academic medical center in outpatient clinics that serviced patients with mTBI. Outpatient visits took place across 7 clinical sites and 3 clinical divisions (sports medicine, neurology, rehabilitation). The clinical teams involved in patient care and recruitment included physicians, athletic trainers, and registered nurses. Of note, the structure and clinical teams varied across clinics (table 1).

**Table 1:** Clinical staff composition and location of clinics involved in BHW study recruitment.

Clinic	Clinical Staff Involved	Locations
General Sports Medicine Clinics	Sports medicine physician, athletic trainer, radiologic technologist, registered nurse	Batesville, Liberty*, Winslow, Green Township, Anderson, Mason, Burnet*
Brain Health Wellness Interdisciplinary Clinics (Neurology/Rehab)	Neurologist/PM&R physician, registered nurse, psychologist, medical assistant, school liaison	Mason, Winslow, Burnet, Liberty*
Brain Health Wellness Interdisciplinary Clinics (Sports Medicine)	Sports medicine physician, athletic trainer, psychologist, physical therapist, school liaison	Liberty*, Mason

Abbreviations: BHW, Brain Health and Wellness; PM&R, Physical Medicine and Rehabilitation.

⁎ Hospital-associated clinics; all other locations are outpatient satellite clinics.

**Fig 1:**
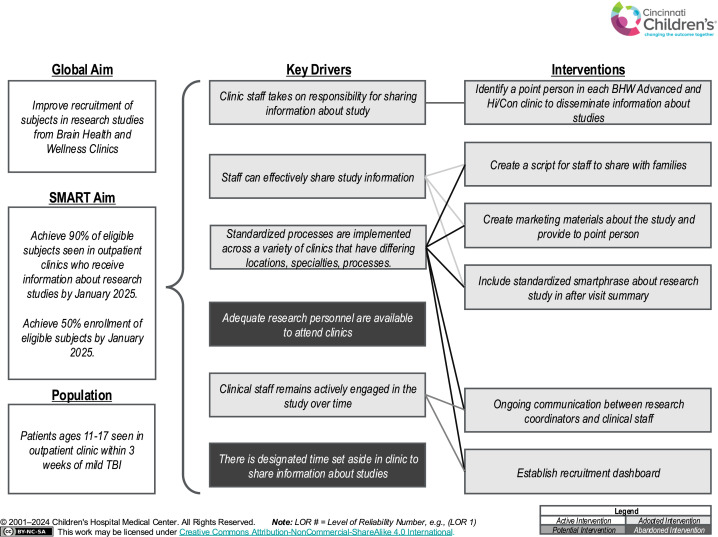
Key driver diagram to improve recruitment in BHW outpatient clinics. Primary aims, key drivers, and interventions addressed in this study. TBI, traumatic brain injury.

The team used a structured QI approach to optimize recruitment in the MOST-mTBI study.^12^ Prior to the start of recruitment, the research team met with the lead author of this QI project, who is a clinician in the Brain Health and Wellness (BHW) clinics, to discuss challenges and key drivers for successful recruitment.^13^ A fishbone diagram was created that identified barriers to success, and the team designed targeted interventions to address key challenges. A key driver diagram^14^ for successful recruitment was created to inform interventions (fig 1). Based on review of the literature and prior experience, the team chose to focus on standardizing communication processes, integrating recruitment into clinical workflows, and leveraging clinical point people (recruitment champions) to enhance engagement.

The QI efforts were initiated prior to the start of recruitment for this study, so the baseline rate was calculated from a prior concussion/mTBI study that focused on school-aged children. Recruitment occurred across the same clinical settings as the current study (eg, outpatient clinics) and the study activities were similar (ie, completion of survey data via virtual platform). As with the present study, clinic staff were contacted and asked to provide study information during a patient’s clinical visit. The baseline study differed in that some children were younger school-aged children; however, most patients seen clinically were in the adolescent age range. To calculate the baseline rate, the proportion of children eligible for the study who were provided with information about the study by clinical staff was calculated across outpatient concussion/mTBI clinics. The baseline rate of eligible patients provided with study information was 32%. Therefore, the primary aim of the present study was to identify key drivers and interventions to improve upon this baseline rate calculated based on similar concussion/mTBI study.

**Fig 2:**
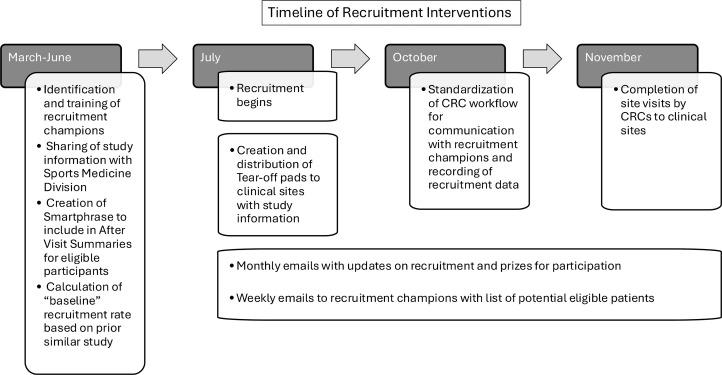
Timeline of recruitment interventions. CRC, clinical research coordinator.

Several small tests of change were initiated at the onset and during study recruitment (fig 2). First, a recruitment champion (typically a nurse or athletic trainer) was identified for each clinic and engaged in the recruitment process. Prior to the start of recruitment, each recruitment champion received information about the study and recruitment goals. In the initial communication and training for the recruitment champions, the research team reviewed the study and outlined expectations, aiming to minimize burden on clinical staff. Expectations of recruitment champions included giving eligible patients study information and reporting back whether they gave this information to each eligible participant seen in clinic.

A standardized workflow was created and followed by the research staff to identify eligible patients. This involved the research team reviewing each clinic schedule in the electronic health record (EHR), identifying potentially eligible patients based on criteria of age and time since injury, and emailing the recruitment champions this list of patients prior to each clinic visit. Templates with study information were provided to the recruitment champions in the form of marketing materials about the study and a smart phrase that could be shared in patients’ after visit summaries so that they had easily accessible, standardized, and accurate information to share with eligible patients. Finally, research staff followed up with recruitment champions via email after each clinic visit to determine if potential participants were informed about the study.

**Fig 3:**
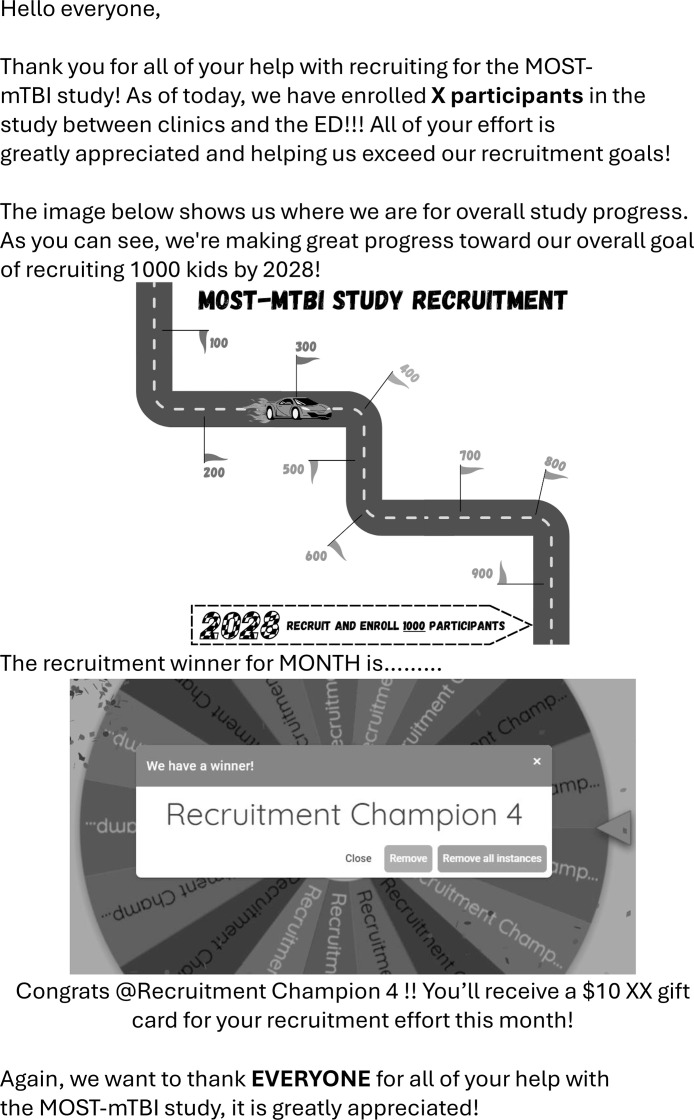
Example template of monthly recruitment update email. Example of an email sent out to all brain injury clinic staff (physicians, nurses, athletic trainers, etc.) each month to (1) provide an update on recruitment and enrollment for the MOST-mTBI study and (2) select a “recruitment winner” based on a lottery system.

To foster ongoing engagement in the recruitment process, the research team planned to send out enrollment updates monthly via email to share progress and offer small incentives (monthly raffle for $10 Starbucks gift cards) that would be awarded to recruitment champions based on a lottery system (fig 3).

Research staff created and maintained a recruitment dashboard to track progress and identify trends in participant engagement. The QI team reviewed and analyzed this monthly, allowing for additional tests of change to be included in subsequent Plan-Do-Study-Act cycles.^15^, ^16^, ^17^ Metrics tracked included the number of eligible patients seen in clinic, the number informed about the study during their clinic visits, and the number who enrolled (with and without receiving clinic information). Chi-square analysis was used to compare proportions.^18^ When a drop in percentage of patients notified in clinic about the research study was seen, a more targeted analysis of which clinics saw the largest changes was performed by further examining metrics by clinic location, clinic type, and department involved. These analyses informed additional interventions including in-person visits to clinics by research staff and obtaining qualitative feedback to help identify barriers to recruitment, assess the feasibility of workflow integration, and refine intervention strategies.

Methods employed for assessing completeness and accuracy of data included comparing recruitment numbers from REDCap exports, research team clinic recruitment spreadsheets, and clinic schedules within the EHR. Missing data arose in this study when recruitment champions did not inform the research team whether participants were contacted about the study during their visit. If recruitment champions failed to provide this information to the research team, those participants were considered to be not contacted in clinic.

## Results

**Fig 4:**
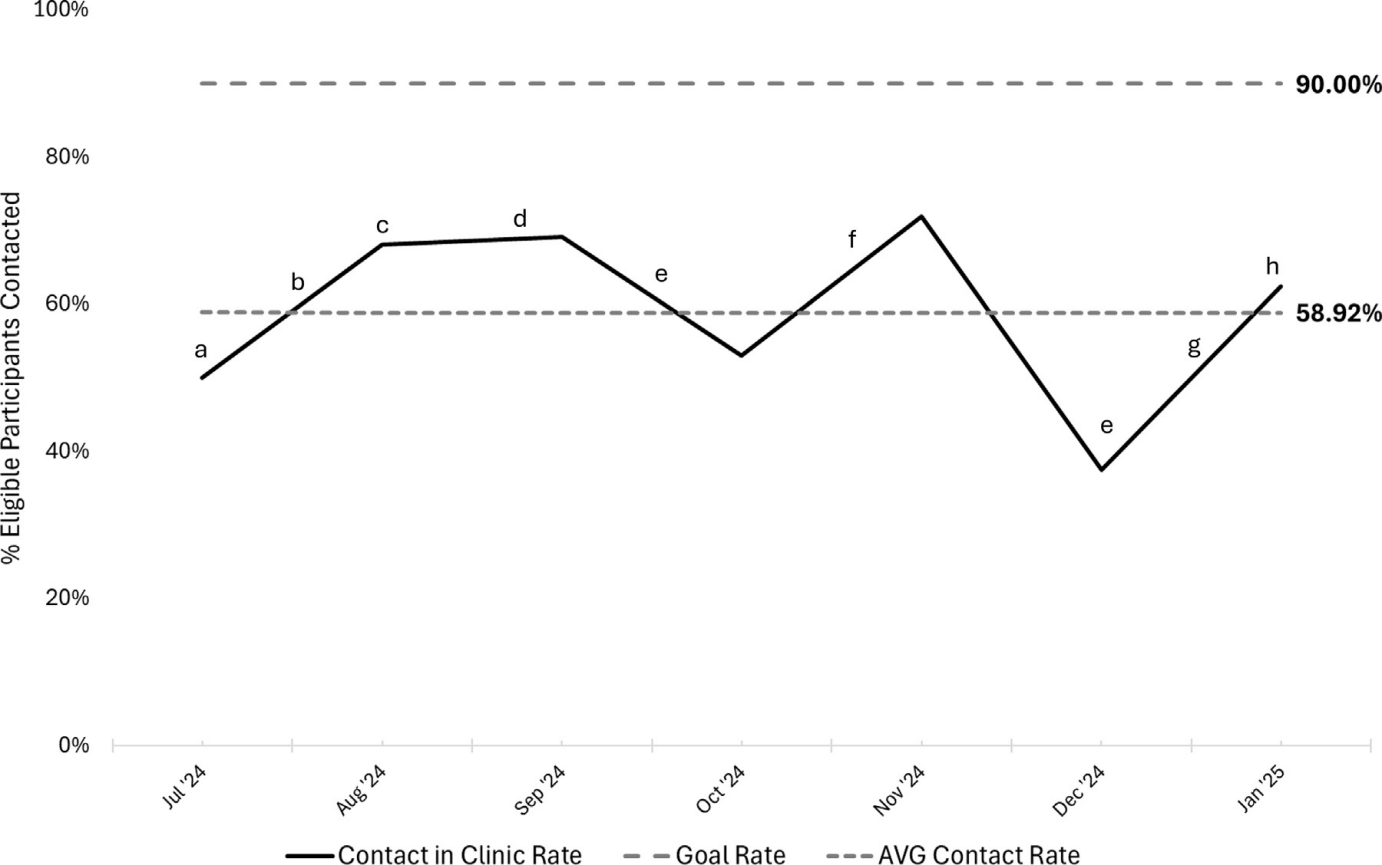
Run chart of eligible patients contacted in outpatient concussion clinics. The percentage of eligible patients seen in outpatient concussion clinics that received study information during their visit from July 2024 to January 2025 (N=231) with annotations for interventions and potential confounders. (a) Recruitment begins in outpatient concussion clinics. (b) Tear-off pads distributed to outpatient clinics. (c) All outpatient clinics have an identified recruitment champion. (d) Standardized recruitment workflow implemented for PMR CRCs. (e) Several point people out of the office across BHW clinic sites. (f) PMR CRCs visit clinic sites to discuss the study and how burden could be reduced for point people. (g) Potentially eligible patients are “flagged” on EHR schedule. (h) Large, laminated marketing posters given to BHW clinics as an additional recruitment material. AVR, average; BHW, Brain Health and Wellness; CRC, clinical research coordinator, EHR, electronic health record; PMR, Physical Medicine and Rehabilitation.

**Fig 5:**
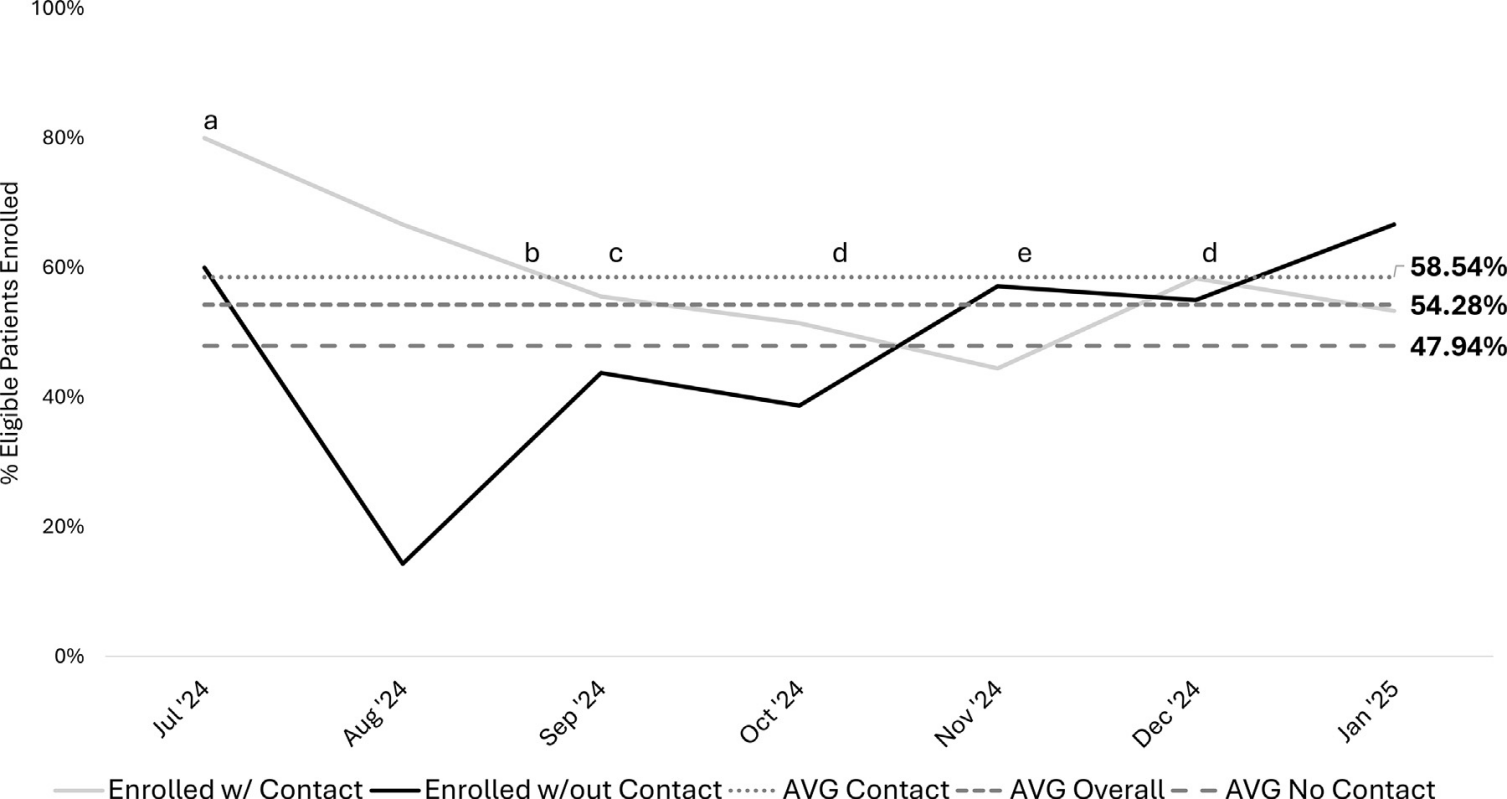
Run chart of eligible patients that enrolled from outpatient concussion clinics. The percentage of eligible patients seen in outpatient concussion clinics that enrolled in the study from July 2024 to January 2025 (N=231), based on whether they received study information during their visit, with annotations for interventions and potential confounders. (a) Recruitment begins in outpatient concussion clinics. (b) Received IRB approval to switch from informed consent to implied consent (interested families can enroll in the study themselves, they do not need to schedule a time for informed consent). (c) Tear-off pad marketing materials are updated with new QR code and link for implied consent (interested families can enroll through QR code/link). (d) Several recruitment champions on out of the office across BHW clinic sites. (e) PMR CRCs visit BHW clinic sites to discuss the study and how burden could be reduced for recruitment champions. AVR, average; BHW, Brain Health and Wellness; CRC, clinical research coordinator; IRB, institutional review board; PMR, Physical Medicine and Rehabilitation.

Over the course of the QI project, a total of 231 eligible patient encounters took place in outpatient clinics between July 2024 and January 2025 (table 2). Recruitment efforts were tracked monthly, and the percentage of eligible patients informed about the study increased from 50% in July at the start of recruitment to a peak of 72% in November. The average contact rate was 58.9% (fig 4). This was significantly higher than the overall recruitment rate of 32% in the prior similar study (χ^2^=36.06, *P*<.001). The study enrollment rate consistently approached or exceeded the target of 50%. Overall enrollment averaged 54% and enrollment from clinic contact averaged 59% (fig 5). Additionally, a correlation was seen between receiving information about the study in clinic and enrollment in the study (χ^2^=21.33, *P*<.001).

**Table 2:** Demographics and characteristics of participants recruited and enrolled through BHW outpatient clinics.

Characteristic (n=99)	n (%) or Mean ± SD
Age (y)	14.1 ± 2.0
Sex	
Male	53 (53.5%)
Female	46 (46.5%)
Race	
White	74 (74.7%)
Black/African American	15 (15.2%)
Multiple race	7 (7.1%)
Prefer not to answer	3 (3.0%)
Ethnicity	
Hispanic/Latino	4 (4.0%)
Not Hispanic/Latino	91 (91.9%)
Unknown	2 (2.0%)
Prefer not to answer	2 (2.0%)
Mechanism of injury	
Organized sport	64 (64.6%)
Fall (ground level)	7 (7.1%)
Motor vehicle accident	6 (6.1%)
Other	6 (6.1%)
Recreational activity	5 (5.1%)
Fall (elevation/stairs)	3 (3.0%)
Object struck head (accidental)	3 (3.0%)
Assault	3 (3.0%)
Motorized transport	1 (1.0%)
Walked/ran into object	1 (1.0%)

## Discussion

The implementation of structured recruitment interventions led to substantial improvements in recruitment rates compared to the prior similar study within outpatient clinics. The findings align with the project’s hypothesis, demonstrating that systematic recruitment strategies can enhance research enrollment rates without compromising clinic flow or patient care. Similar to prior research, a multipronged approach to clinical recruitment was required.^5^

We did not achieve our aim of providing study information to 90% of eligible patients in clinics, and several learning points emerged for our team during this QI process. A notable benefit was the improved communication and collaboration between clinical and research teams, allowing for a streamlined recruitment process. Additionally, the structured approach to identifying “recruitment champions” fostered a sense of ownership among clinical staff, leading to greater engagement in research recruitment.

There was monthly variability in patient contact throughout the study (fig 4). Clinic staff schedules and clinic workflows all affected patient contact. For example, some designated recruitment champions had extended leaves during the recruitment period leading to cross-coverage by different clinical staff who were not familiar with the study. This likely contributed to reduced contact rates during these time periods. Additionally, recruitment champions shared that during clinics with higher patient volumes, challenges with workflow and clinical burden made the introduction of study information to patients more difficult and less consistent. Although the goal was to minimize burden for the clinical staff associated with sharing study information, during periods of short staffing and/or increased patient volumes, the burden was amplified. Given the relatively predictable increase in volume of patients seen with mTBI during fall sports seasons, we plan to preempt disruptions during upcoming busy seasons by implementing regular in-person check-ins at specific intervals. For the upcoming recruitment year, we intend to have research coordinators perform in-person site visits in spring and early fall. These visits will serve multiple purposes including gaining information about expected time off and potentially identifying interim recruitment champions, reminding clinical teams about the research study ahead of busy clinical seasons, and anticipating and mitigating potential barriers to providing study information prior to the anticipated increase in patient volumes.

A key strength of this QI study was the successful collaboration between clinical and research teams, which facilitated a more sustainable approach to recruitment. This collaboration within an interdisciplinary team is a familiar model in rehabilitation but could certainly be implemented in recruitment across specialties. Identifying and training clinical recruitment champions proved to be an effective strategy for embedding research activities within existing clinical workflows. Additionally, the use of multiple recruitment modalities—ranging from preclinic email notifications to in-person engagement and follow-up messaging—allowed for a more comprehensive approach. Recruitment champions reported that they felt engaged in research activities, and the structured approach reduced the logistical burden of recruitment.

These findings align with prior research emphasizing the importance of active, systematic recruitment strategies in clinical settings. Previous studies have shown that passive dissemination of research information is insufficient to drive enrollment and that structured, team-based approaches improve recruitment outcomes.^1^ The use of clinical staff and designated research staff for recruitment has been reported to be an effective method in similar studies,^6^ further supporting the success of the strategies implemented in this project.

The implementation of this project required modest resource allocation, primarily in training efforts and incentive programs for clinic staff. The introduction of small incentives, such as gift cards for engaged clinical point people, was a cost-effective approach to maintaining motivation. However, the team building and camaraderie created from these activities may have had greater impact than the monetary incentive.

### Study limitations

This study solely involved recruitment of adolescents with mTBI within outpatient concussion clinics at one institution. Further research is needed to evaluate these same practices within settings such as inpatient, emergency department, urgent care, and other community settings. Additionally, many rehabilitation populations have limitations in mobility as well as other physical and psychosocial barriers that may present challenges to participation in clinical studies that were not faced in this study.

The interventions used in this project were restricted to the outpatient clinic setting. Community-based and behavioral approaches to recruitment and retention can be considered in future work.^19^^,^^20^ Confounding variables, such as variability in clinic staffing and clinic workload may have impacted the effectiveness of the interventions. Additionally, the self-reported nature of data collected from recruitment champions may have introduced reporting bias, as some staff may have inconsistently documented their recruitment efforts. As part of our effort for maintaining ongoing engagement from recruitment champions, we used small gift cards as rewards for participation in recruitment. We believed the value of the gift was low enough to not be ethically concerning and was equivalent to what a colleague might give as a thank you to another colleague for help with a work-related task.

## Conclusions

This project demonstrates the application of QI methodology toward optimizing clinical recruitment. Developing research enrollment processes that integrate with clinical processes improved the rate of patients receiving information about the study and led to improved enrollment rates. Identifying clinical team members to be the recruitment champions in clinical areas was successful. Overall, this project provides support for the use of QI and implementation science methods as tools to optimize enrollment in clinical research studies. Approaches used in this study can potentially be generalized to other outpatient clinical populations and research studies.

## Disclosure

The investigators have no financial or nonfinancial disclosures to make in relation to this project.
